# Reconfiguring health knowledges? Contemporary modes of self-care as ‘everyday fringe medicine’

**DOI:** 10.1177/0963662520934752

**Published:** 2020-06-27

**Authors:** Pia Vuolanto, Harley Bergroth, Johanna Nurmi, Suvi Salmenniemi

**Affiliations:** Tampere University, Finland; University of Turku, Finland

**Keywords:** health and new technologies, lay expertise, patients, public understanding of science, science experts, studies of science and technology

## Abstract

The contestation of expertise is perhaps nowhere more pronounced than in the field of health and well-being, on which this article focuses. A multitude of practices and communities that stand in contentious relationships with established forms of medical expertise and promote personalised modes of self-care have proliferated across Euro-American societies. Drawing on multi-sited ethnography in three domains – body–mind–spirit therapies, vaccine hesitancy and consumer-grade digital self-tracking – we map such practices through the concept of ‘everyday fringe medicine’. The concept of everyday fringe medicine enables us to bring together various critical health and well-being practices and to unravel the complex modes of contestation and appreciation of the medical establishment that are articulated within them. We find three critiques of the medical establishment – critiques of medical knowledge production, professional practices and the knowledge base – which make visible the complexities related to public understandings of science within everyday fringe medicine.

## 1. Introduction

Sociologists of science have long been drawing attention to the changing status of expertise in contemporary societies (Collins, 2014; Wilcox, 2010; Wyatt et al., 2010). Although expertise and experts continue to enjoy high regard and trust in a number of spheres, contemporary media and particularly the Internet have crucially transformed the ways in which expert knowledges are constructed and claims to expertise are made. A host of new ‘cultural intermediaries’, such as life coaches, food bloggers and lifestyle gurus, populate the media landscape and offer guidance on issues of health and well-being. Such forms of ‘lay expertise’, often drawing on experience-based expertise, have become increasingly influential in various arenas of social life (Wilcox, 2010). Harry Collins (2014; see also Collins and Evans, 2002) has pondered whether the apparent loss of public trust in scientific expertise means that more or less ‘anything goes’ in the contemporary expertise game, and some have even gone as far as envisioning the ‘death of expertise’ (Nichols, 2017). Such accounts signal intensified struggles over what counts as expertise and who can be an expert.

The contestation of expertise is perhaps nowhere more pronounced than in the field of health and well-being, on which this article focuses. A multitude of practices and communities that stand in a contentious relationship with established forms of medical expertise and promote personalised modes of self-care have proliferated across Euro-American societies. Often such practices have been captured through the concept of ‘complementary and alternative medicine’ (CAM), typically presented as ‘the other’ of the Western biomedical paradigm. However, as several commentators have pointed out, the concept of CAM and its many related terms, such as natural and traditional, are controversial (Barcan, 2011; Gale, 2014; Louhiala and Puustinen, 2012; Saks, 2003) in that they tend to connote a polarised understanding in relation to biomedical knowledge. Critique is rarely simply ‘against’ biomedical knowledge, but is rather a complex and even paradoxical mess of ideas pertaining to morally proper and individualised modes of health-related knowledge production (see Jauho, 2016). As we will highlight in this article, many of the everyday self-care practices cannot be unambiguously categorised as either merely ‘alternative’ or ‘biomedical’, but they rather destabilise and negotiate the alternative-biomedical boundary in many ways.

This article tackles questions of expertise and health-related knowledge by drawing on multi-sited ethnographic research into the ways in which users of different self-care practices perceive and encounter the ‘medical establishment’ – medicine, medical knowledge, medical expertise and health authorities. At the heart of our investigation lie the following questions: *What rationalities and claims to expertise underlie engagement in these self-care practices? What relationships to the medical establishment are constructed in them?* The purpose of the article is twofold. First, we make a theoretical contribution by introducing the concept of ‘everyday fringe medicine’ (EFM). This concept allows us to respond to the critique levelled at the concept of CAM and to capture the complexity of contestations of the biomedical paradigm in everyday self-care practices. Second, we illustrate this complexity by introducing three forms of critique of the medical establishment articulated within EFM that show how EFM practices *both* challenge *and* collaborate with biomedical modes of health expertise and science.

By addressing these questions, the article contributes to the literature on the public understanding of science and the sociology of health expertise in three ways: first, by developing the novel conceptual tool of EFM to theorise the logics of everyday self-care practices and their relationships to medical knowledge and practices; second, by empirically highlighting the complexity of critiques of the medical establishment among EFM users and third, by making visible the complexities related to the public understanding of science. While in public discourse many forms of the lay appropriation of medicine continue to be framed as ignorance or misunderstanding of medical science, the modes of critique and contestation evident in EFM direct the gaze towards the social shaping of epistemic authority by elucidating various modalities of engagement with the medical establishment.

The article will proceed as follows. We will first outline the way in which our conceptual and empirical arguments developed during the course of a research project on self-care practices. We will then relate our research to sociological discussions of ‘alternative’ health practices and introduce in more detail the concept of EFM as a conceptual aid for investigating the multiple relationships with the medical establishment in contemporary health landscapes. In the subsequent sections, we will illustrate three forms of critique emerging in our ethnographic materials, before providing conclusions.

## 2. Ethnographies of self-care

The research materials for this study come from the research project *Tracking the Therapeutic: Ethnographies of Well-Being, Politics and Inequality* (2015–2019), which investigated a variety of self-care practices in Finland. Taking an ethnographic approach, the project uncovered how and why people engage with everyday self-care practices and how they make sense of and experience them (Marcus, 1998). It also interrogated the multiple forms of knowledge that people produce, synthesise and mobilise in caring for themselves and those close to them. In this article, we focus on three self-care practices studied on the project: body–mind–spirit practices, vaccine hesitancy and self-tracking. We thus address a spectrum of contemporary modes of self-care ranging from ‘holistic’ and ‘natural’ practices to technoscientific practices and ‘hip’ consumer health technologies.

Finland offers an interesting case for studying evolving relationships to the medical establishment, for several reasons. Finland is a Nordic welfare state with a strong public healthcare system, where there is a strong trust in medical authorities and science institutions (Finnish Science Barometer, 2019). The boundary between official medicine and its ‘alternatives’ is sharp. CAM is not officially recognised, and unlike in most Nordic countries, there is no legislation to regulate it (CAM Regulation, 2013). The discussion around different modalities of treatment is highly polarised. While around 30% of Finns have used some form of CAM (Kemppainen et al., 2018), these treatments tend to be viewed with suspicion by medical professionals and authorities, as demonstrated, for example, in the term ‘belief medication’, promoted by the Finnish medical professionals’ association (Finnish Medical Association, 2017).

Fieldwork on body–mind–spirit practices targeted both professional healers and people who performed the practices as part of their everyday self-care. Roughly half the research participants worked as full-time or part-time healers. However, the boundary between healers and those who perform body–mind–spirit practices as part of everyday self-care is blurred, since professional healers also engage in them in their own everyday self-care, while ‘non-professionals’ occasionally administer these practices to their friends or family members free of charge. We can thus conceive of them all as ‘practitioners’ in the sense described by Thomas McLaughlin (1996: 22), who suggests that those who practise a given craft or skill always develop a ‘vernacular theory’ of their practice, that is, how it ought to be practised and the values, concepts and worldviews associated with it. The research participants were identified through the Internet and at a range of public events, as well as through a snowballing technique. All of them engaged with a wide array of practices, including mindfulness, reiki, life coaching, angel healing, yoga, art therapy, self-help reading, folk healing, acupuncture, reflexology, aromatherapy, astrology, herbal medicine, homoeopathy and many more. The research materials include interviews (*n* = 32, 30 women and 2 men), media materials (webpages, popular books, newspaper columns, etc.) and participant observation at a range of body–mind–spirit events. The interviews explored the practitioners’ experiences of and motivations for engaging with body–mind–spirit practices, the role these practices played in their everyday lives and in society more generally, their perceptions of and encounters with official medicine, and their political views and engagements.

Fieldwork on vaccine-hesitant families included ethnographic interviews with parents of partially vaccinated or non-vaccinated children (*n* = 33, 31 women and 2 men) and observations on social media. Participants were mostly reached through a vaccine-critical open Facebook group. Later, those who had already participated also referred more participants to the study. The participants had opted out of all or several of the recommended vaccines for at least one of their children. Some had fully vaccinated their older children before starting to question vaccination. The participants had a total of 97 children, aged between 23 years and 2 months. Of these children, 46 were unvaccinated, 38 were partially vaccinated and 13 were fully vaccinated until at least the age of 6 years. The interviews covered three major themes: the experiences and reasons that had led participants to question vaccination, their health attitudes and practices and their encounters with healthcare professionals. Participants were asked where they sought information about vaccines and how they evaluated the information they found from different sources. They were also asked whether they trusted medical research about vaccines.

Fieldwork on self-tracking included interviews (*n* = 19, 7 men and 12 women) with people who had recently conducted voluntary, proactive self-tracking using one or more consumer self-tracking devices, such as activity tracker wristbands, sleep-tracking devices, heart rate monitors and laboratory measurements. It also included media materials, marketing materials on self-tracking devices, observations at digital health-related events in Finland and observations of discussions in a Finnish self-tracking-related Facebook group (‘Quantified Self and Biohacking Finland’). Research participants were reached both through the abovementioned Facebook group and through people who had no known contact with such groups. Thus, the participants represented a heterogeneous group, as some were enthusiastic self-trackers or early adopters, while others had very limited experience with such technologies. The interviews covered their motivations for and experiences of self-tracking, and how they viewed the significance and future development of self-tracking. Participants were not explicitly asked about their relationship with official or alternative modes of health knowledge, but these themes emerged spontaneously in the course of the interviews. Most research participants did not express explicit doubts about science or medicine and used the devices to ‘stay in shape’ according to expert guidelines. However, more experienced self-trackers mentioned their desire to employ self-tracking as a mode of personal data analytics.

Our analysis focuses on the interview narratives and media materials, with ethnographic observations providing contextual sensitivity and background information for the interpretative work. The research materials were analysed using qualitative content analysis driven by the theoretical focus of the article. The analysis first centred on the boundaries and hierarchies between EFM and biomedicine (both medical practice and medical research) described by the participants. In the subsequent rounds of analysis, we traced the participants’ relationship to biomedical research and knowledge, as well as their engagements with the medical community and health authorities. We discovered that despite the apparent differences in the three self-care practices we addressed, similar themes and arguments kept surfacing regarding the medical establishment and especially scientific medical expertise. Moreover, in all these sites, the arguments tended to reflect a notable interest in and multifarious attachments to medical science and biomedicine, instead of simple ignorance or hostility. This led us to examine in more detail what these different health practices could reveal about the changing and tumultuous social terrain of health expertise. By bringing together the research materials gathered during fieldworks, our ultimate aim was to relate these sites of self-care to each other and flesh out ways in which EFM users made sense of their relationship to biomedicine, medical practices and encounters with medical personnel. When thinking about how to conceptualise the different forms of self-care in relation to the social construction of *expertise*, we felt restricted by the existing conceptual frameworks. Discussions of ‘complementarity’, ‘alternative health’ or counter-expertise did not seem fitting to describe the complexity of the practices or their critiques of medicine. We then arrived at the concept of EFM, through which we were able to tie together these different domains of self-care and their interaction with medical science and biomedical modes of thought and action.

On the basis of our analysis, we identified three forms of critique: (1) the critique of medical knowledge production, (2) the critique of medical professional practices and (3) the critique of medical experts’ knowledge base. It is important to note that these different types of critique are not mutually exclusive but overlap in many instances. The point here is not that all practices reflect all these forms of contestation equally (they definitely do not), but merely that all of these practices work as everyday regimes of self-care in which contestation (or acceptance) of medical expertise is negotiated. In our analysis, we have aimed to present the forms of critique in a succinct manner and illustrate how critique towards health knowledge promotes both evaluation of and engagement with medical knowledge or the medical establishment. Our main contribution relates to the concept of EFM, which we suggest can be employed to further research on both ‘traditional’ and emerging forms of health-related knowledge production in the context of public understanding of science and expertise. The empirical illustrative analysis in this article serves to underline this point.

## 3. From complementarity to EFM

Traditionally, the term ‘fringe medicine’ has encompassed a wide range of therapies and health practices, such as herbal remedies (Evans, 2001) and hydropathy (Peeters, 2010), that are situated ‘at the fringes’ of official or generally accepted forms of healthcare. However, the concept is seldom used compared with the more familiar concepts of ‘complementary’, ‘alternative’, ‘traditional’, ‘quack’ or ‘irregular’ (on these concepts, see Gale, 2014). Studying CAM, Derkatch (2016: 7) suggests the terms ‘fringe patients, fringe illnesses, fringe practitioners and fringe health models’ to describe broad means of caring for one’s own health which ‘fail, somehow, to fit within the accepted boundaries of mainstream scientific medicine’. Inspired by this elaboration, we suggest that the term ‘fringe medicine’ can capture the multiple traditional and modern forms of self-care practice that critically *engage with* biomedicine or can be situated at the *boundaries* of medicine and medical practice. To this concept, we have added the temporal dimension of ‘everyday’ to emphasise that such self-care practices often constitute a routine part of daily life. Of course, in common sense thinking, it might be argued that whereas a mode of self-care, such as vaccine hesitancy clearly ‘does not fit’ with the biomedical paradigm, the data-oriented rationality of tracking and analysing one’s activity or sleep with digital gadgets in many ways does. However, even the latter form of everyday self-care is most often labelled – by technology developers, users and medical instances alike – as ‘non-medical’ technology, and often, for various reasons, as inadequate on its own for the individual to really understand their health and well-being. In this sense, self-tracking too can be argued to occupy the biomedical fringe. The crucial point, however, is that although they take a critical stance, none of these practices seek to entirely abandon or reject biomedicine or science-based knowledge.

The important question, then, is not the extent to which EFM practices comply with, resist or even reject dominant medical practices by adopting non-normative health behaviours, as some previous research has outlined (e.g. Keshet and Popper- Giveon, 2018), but rather how these forms of everyday health behaviour ‘mix and match’ biomedical and alternative modes of knowledge and practice in acting on and knowing about one’s health and well-being. At the heart of EFM is the acknowledgement of its contradictory character; the ways in which it *both* aligns itself with biomedical expertise *and* seeks to transform it. For us, the term EFM highlights how self-care practices build not only on the contestation of scientific expertise but also on the appropriation and acceptance of medical science; not only on the rejection of biomedical knowledge but also on active interpretations of and ‘working with’ medical knowledge and scientific or evidence-based modes of knowledge production on health and illness.

Thus, EFM allows us to capture a broad set of practices and groups that share certain values, traditions and modes of knowing about health and well-being that appear to challenge biomedical practices in some ways while also supporting them in other ways and suggesting new modes of collaboration with them. Importantly, while the term ‘alternative’ denotes exclusion from that which is hegemonic (Barcan, 2011; Gale, 2014), ‘fringe’ focuses on that which is on the edge, boundary or margin (or maybe marginalised). EFM is not ‘complementary’; sometimes it is almost fully aligned with biomedicine, and usually it is somewhere in the grey boundary zone between many intersecting worlds. Thus, unlike the concept of CAM, EFM does not assume a medical starting point and does not position any health practice a priori as being on the ‘outside’ (due to lack of evidence, for example); rather, it adopts a novel, social scientific point of departure, especially in relation to the growing contestation of expertise. While the concept of ‘fringe medicine’ could be perceived as value-laden and pejorative, we do not mean to employ it as such. Instead we wish to underline the constant negotiation and co-creation of expertise by highlighting the ways in which those ‘at the fringe’ engage with and negotiate medical scientific knowledge production and professional practices. Taking a cue from the call in social studies of science and expertise to pay close attention to the formation of systems of knowledge with an open mind (Harding, 2008; Wilcox, 2010), our analysis addresses the ways in which users of EFM assemble health knowledges by relating to knowledge production in medicine (medical research), expert knowledge (the work of physicians and other professionals) and official health recommendations (e.g. by state authorities).

Previous research on the public understanding of science pertaining to groups that are critical of the medical establishment has shown that in public discussions and academic circles alike, many forms of the lay appropriation of scientific claims still tend to be framed as public ‘misunderstandings’ (or ‘ignorance’) of science or constructed as ‘the other of science’ (Goldenberg, 2016; Harambam and Aupers, 2015). Such work evinces that despite discussions since the mid-1990s of the inappropriateness of the deficit model – that is, the tendency to see the public as ignorant or poorly informed – there is still more to explore in the understanding of science among its publics (Goldenberg, 2016; Harambam and Aupers, 2015; Jauho, 2016; Stocking and Holstein, 2009; Wynne, 1995). In line with this research, we argue that the deficit model obscures how EFM users critically and selectively engage with and negotiate medical science and expertise. Similarly to Harambam and Aupers’s (2015) study of conspiracy theorists, our purpose is not to assess the truth value of EFM epistemologies but rather to highlight the social processes through which the relationship to scientific knowledge and expertise is built up in EFM.

Quite early on in our research, we noticed that EFM practitioners did not perceive anything a priori wrong or suspect about pursuing medical knowledge through scientific means. Rather, the critical arguments revolved around moral, humanistic, epistemological and evidence-based rationalisations of how scientific knowledge production, medical professional practices and the formulation of the medical knowledge base should work ‘better’. In the following sections, we will lay out three forms of critique present in our research materials to demonstrate EFM users’ rationalisations of health practices, as well as the intersections of such rationalisations among the three EFM domains we studied. By so doing, we will illustrate the ways in which practitioners of EFM seek to both challenge and collaborate with the medical establishment. The analysis is not meant to compare different EFM domains or to set up stark contrasts between them. Rather, our goal is to demonstrate the analytical purchase of the conceptualisation of EFM by teasing out the discursive strategies and rationalisations through which all these practices may be thought to hover at the fringe of biomedicine.

## 4. Critique of medical knowledge production

The first form of critique centres on medical knowledge production. It addresses most notably the logic of (medical) capitalism, and more specifically the idea that health is subjugated to market logic and profit. Research participants were sometimes deeply concerned that economic interests dictated research, treatment and the overall politics of health, which rendered medical science and expertise unreliable or suspect, if not downright corrupt. This critique conjured an image of a ‘biomedical complex’ through which economic, political and medical interests intertwined and reinforced each other.

The participants considered that a substantial amount of medical research on the safety and effectiveness of pharmaceutical products was biased, flawed or distorted. They were concerned about the negative side effects of medications and vaccinations and argued that such products were pushed onto the market, despite their known side effects. In their view, biomedical research failed to meet the moral and ethical standards required of the field, and medical knowledge often took the form of corporately induced ignorance. A typical line of argument was summarised by Jenny, a vaccine-hesitant parent, who argued that ‘scientific data is a bit questionable because what is being researched, what the hypotheses are and how research is being done is tied to money’. Irene, the mother of an unvaccinated child, echoed this by saying that pharmaceutical companies could confirm the results they wanted by doing choice work on how they delimited or cropped the data and which differences or significances they chose to highlight. A typical suggestion for the transformation of medical research in such critiques was to point out the ties that researchers or research funders might have, or to rationalise and cite examples of the ways in which research might have been distorted by the picking and choosing of results or data. Another major concern was the perceived distortion or corruption of medical science due to the dominance of pharmaceutical companies in the funding and conduct of biomedical research. Hanna, a body–mind–spirit practitioner, argued,

They just medicate and medicate in order to barely keep you alive, and then you need more drugs, since there are always side effects, and of course it’s good that they can keep you alive because then they can sell you more drugs.

Part of the critique was also targeted against the biomedical paradigm as ‘the only game in town’ (Barcan, 2011), that is, the only form of evidence and expertise that is recognised. The participants complained that if EFM knowledge or experiences did not fit this paradigm, they were rendered invisible and deemed irrelevant. This was summarised by Pia, an entrepreneur in her 40s who practised body–mind–spirit techniques:

There’s so much alternative care and knowledge available, including research, but because it is not medical research it is not accepted in the healthcare system. Why? Because they just keep repeating that it’s not medically proven, although there’s loads of studies on functional medicine that have shown, for example, that milk is not good for people. . . . But because these are not biomedically studied, they do not exist.

It is important to note that even those who were most explicitly critical of medical capitalism typically did not reject medical research or science as such, but rather problematised the funding of clinical research and expressed concern over how vested economic interests shaped health policies and care practices. In their view, scientific knowledge should be free from vested interests – a critique directed at the ‘purported neutrality or objectivity of scientific research’ (Harambam and Aupers, 2015: 473). Of course, the critique that medicine has been commodified, and that health has become subject to the capitalist logic of profit-making, is not new; it is a long-standing critique in both holistic health movements and the social sciences (see e.g. Dumit, 2012; McKee, 1988). What is interesting today is how such critique takes shape in relation to emerging contemporary and technoscientific forms of everyday self-care.

While in interviews with self-trackers this type of critique was not explicit, and many self-trackers expressed strong trust in the medical establishment, some participants did draw links to ‘biohacking’, referring to discourses about approaching one’s body as a system to be ‘hacked’ in the original (positive) sense of the word, that is, tweaked and improved by tinkering with it on one’s own terms. Interestingly, the local Finnish manifestation of the global ‘Quantified Self’ movement (see Lupton, 2016) – Quantified Self and Biohacking Finland – has in recent years been pioneered and personified by a trio that consists of a technology entrepreneur, a nutrition expert and a medical doctor who is a practitioner of functional medicine. They have all appeared as speakers in numerous health-related events and together have published the popular non-fiction book *Biohacker’s Handbook* (Arina et al., n.d.), and their public performances have reflected a tense relationship with biomedicine-based knowledge in multiple ways. For example, the biggest Finnish daily published a piece on biohacking in November 2013 where one member of the trio told having self-cured a stress-based ulcer (Frilander, 2013). He had achieved this by first reading through hundreds of scientific articles and then developing a systematic personal programme that involved tracking a multiplicity of biomarkers with consumer-grade self-tracking technologies and using private laboratory testing services because all one could get from official healthcare was medicine that seemed to help only for as long as it was taken. Here the ‘work’ done with medical research involved reading medical literature and using it to design a regime of personal self-care that built on lifestyle changes and nutritional choices instead of medicine. So in biohacking and self-tracking discourse, medical science is revered, but self-tracking may become constructed as a sociotechnical domain of self-care that enables personal ‘science-based’ knowledge production while also incorporating discourses of suspicion towards medicine or medical products. It can convert into full-blown moral critique against corruption if official (health) information is perceived as misleading or skewed. Such moral critique was also articulated by another member of the biohacking trio, who claimed that official Finnish nutrition guidelines are designed to serve the interests of local food industry (Simola, 2013), a critique often voiced by body–mind–spirit practitioners and vaccine-hesitant individuals, too.

The main object of this form of critique, then, is the field of medical and health-related science and expertise in their sociopolitical context, often pointing to the corruptive influence of medical capitalism. In this sense, the critique points to the ‘invisible hands’ (Sismondo, 2018) of pharmaceutical industries and other commercial actors behind biomedical knowledge production, which is one of the common ways in which health practices are positioned at the ‘fringe’. This ‘corrupt’ and/or capitalist logic of health may be abandoned or ‘rejected’ in everyday practice by deliberately adopting a stance of opposition through the selective utilisation of biomedical products. For example, some of our vaccine-hesitant families and body–mind–spirit users explained that they might consult a medical doctor to receive a diagnosis but then treat the condition with EFM, for example, by homoeopathic methods. Furthermore, some body–mind–spirit therapy users and self-trackers talked about gathering data – by analogue and digital means, or, for example, using consumer services for laboratory tests without a doctor’s referral – and then doing interpretative work, seeking to balance their health through a personalised assemblage of medical and EFM knowledge. These examples highlight how EFM practices draw on many knowledge regimes and practices, not only medical, although biomedicine usually remains sometimes in the background, sometimes in the foreground and at least as a safety net in case all else fails.

We argue that EFM users in no way deem science in general or medical science, in particular, to be useless or irrelevant (see also Harambam and Aupers, 2015: 473). Rather, and similarly to the findings of studies of lay perceptions of medication (Webster et al., 2009), they maintain that biomedical research and practice should ensure patient safety and be based on efficacy, and that the avoidance of side effects should be stressed more than is currently the case. The medical establishment, in the form of scientific literature and/or expert knowledge, should be developed through both the idea of personalised self-care and the attempt to ‘purify’ it by challenging medical capitalism. In this critique, although participants are taking issue with the practices of medical knowledge production, they are also expressing a desire to participate in the articulation of the ethical and moral principles of research and health knowledge. They demonstrate their desire to engage in defining the practices of scientific research and especially in delineating its responsible conduct. They also strive to explore and politicise the consequences of the commercialisation of research. Thus, while being a way to challenge and criticise medical knowledge production, this critique also highlights the possibility that EFM users’ concerns may have gone unrecognised by the medical establishment (Wynne, 2006: 219).

## 5. Critique of professional practices

The second form of critique targets the bio-reductionist professionalised medical expert system, the thrust of the critique being medical professionals’ unwillingness and inability to ‘meet people’ as emotional, spiritual and communicative human beings. It also partly reflects a more general perception of the medical (scientific) expert system as exclusive, unreachable or too bound up with its own impersonal and mechanistic ways. For many, it is not just medical treatments and medications that have healing power and constitute care but also, very importantly, communication and embodied encounters between patients and health professionals. The participants criticised health professionals for not being capable of, or not being allowed to, ‘connect’ on a personal or emotional level and acknowledge emotions as a significant part of human life and health. While many readily acknowledged that a visit to a doctor was often necessary, they harboured doubts about the system’s capabilities to care for or heal them. Despite these participants’ critical views, in practice, there seemed to be a tendency similar to that found by Attwell et al. (2017): even the most vociferous critics of the medical establishment relied on medical knowledge in some instances, such as with broken bones, but they tended to seek emotional and bodily healing encounters elsewhere, in the EFM domain.

Many participants proposed that medical professionals should fulfil their function of care in society by taking emotions seriously, as well as by acknowledging personal experience and experiential knowledge as crucial resources for successful care. The participants recounted incidents when medical personnel and experts had been blasé, indifferent and (too) ‘professionalised’ in relation to people’s own experiences and systems of knowledge. Such critique typically identified medical training as a root cause of this. Medical training was seen as overlooking the emotional and spiritual dimensions of health and illness, and as leaving medical professionals unwilling or unable to acknowledge patients’ needs, experiences and competing knowledge claims. Isabel, a vaccine-hesitant mother, explained this point:

The problem is, if you go see a doctor or a nurse and you say that we really suspect that we’ve experienced adverse effects from a drug or a vaccine. But they firmly believe in what they’ve been taught. So they judge you very easily. They don’t even let you finish talking. And that suppresses all discussion.

Nora, a body–mind–spirit therapist argued that ‘what really makes these treatments effective is the therapist’s presence to the client, that the client can feel that, for once, she gets seen, heard and accepted’. Nora attributed a crucial healing effect to this recognition and compassionate validation of the client’s experiences’ (see also Sointu, 2006).

Among self-trackers, it was typically recognised that medical personnel may or may not be open to people’s own deductions about actual or potential health threats, or about states that people had monitored or observed in their data. For example, it could be perceived that doctors can be somewhat uneasy about letting patients step into their territory of expertise. Also, as is typical in biohacker discourses more generally, while medical expertise was typically highly appreciated, participants sometimes implied that doctors treated illness mechanically, instead of really adhering to the complexities of well-being. For example, Jari, who had been rigorously measuring various aspects of his life for years, and whose motivation to self-track was now mainly to conduct an ongoing ‘expedition into himself’, clearly held medical expertise in high regard, but also pointed out that there was now a ‘big discussion’ about well-being in the sense that various events and seminars explore different dimensions of well-being grow in popularity. He highlighted the meaning of happiness for well-being said there was no straightforward definition of well-being, although ‘doctors might say that well-being is the absence of a diagnosed sickness’. Especially in relation to preventive care, such articulations imply that holistic and ‘alternative’ influences work to shape self-tracking into a practice that opens up possibilities both to support biomedical knowledge production and to criticise the expert system as too professionalised or too set in its own ways.

This critique resonates with the discourse of ‘personalised healthcare’ (Topol, 2015; see also Harris et al., 2010; Sharon, 2017), which encourages health-related participation, proactive action and self-awareness. Personalised medicine is often presented and promoted as a field of collaboration and partnership between official medical professionals and actual or potential patients. However, with its emphasis on individual action and responsibility for one’s own well-being, it can also be both reflective and formative of varying degrees of critique of medical expert systems. As has also been argued for CAM modalities, for EFM users holism may act as a form of recognition of the multiplicity of emotions, thoughts and lifestyles (Barcan, 2011: 25).

In this critique, the existence of a professionalised medical expert system is not questioned; rather, it is seen as inflexible, wrongly calibrated or incapable of performing its function in society because it is reductionist instead of holistic, or not open to dialogue. The critique is thus not primarily against the expert system as such, but points to EFM users’ concerns about failures in emotional and embodied communication practices, lack of recognition and/or society’s inadequate acceptance of personal experience as a crucial resource for fulfilling care functions. On the other side, these concerns may be interpreted as a willingness to further develop the good practice of medical professionals. Also, it can be highlighted that EFM users’ focus on assessing professionals’ communicative practices points towards important interconnections between trust and dialogue that may have gone unrecognised by the medical establishment (Goldenberg, 2016: 574).

## 6. Critique of the knowledge base

The third type of critique focuses on experts’ knowledge base, which consists of population-level recommendations and evidence-based guidelines. It stresses the significance of personal evidence and the individual’s personal knowledge production alongside population-level generalisations. The participants employed a variety of methods from personalised experience-based or data-based knowledge that for them constituted evidence.

Our research participants often expressed a willingness to experiment on their own lives and bodies, and even to act as a ‘scientist of one’s own life’, as one of the self-trackers put it. They were often keen on experimenting, for example, with various body–mind–spirit therapies, nutritional choices, exercise patterns, creative practices (singing, dancing, painting etc.) and other behavioural changes to improve their health and well-being and on seeking to ‘validate’ their experiences of the effects of such choices through self-compiled data (in the context of self-tracking as a research-like activity, see Heyen, 2020). For self-trackers, digitally compiled data enabled a possibility to investigate, for example, how changes in everyday habits affect sleep, recovery or blood pressure. In the body–mind–spirit sphere, an alternative therapist Marcus had ‘tested’ flower remedies on himself and his dog, searching for evidence that these remedies ‘really worked’:

I have used these remedies and experimented with them. The first case where I clearly saw the effect was my Australian terrier. She had a urinary tract infection that had been treated with antibiotics and homeopathy, but they didn’t help. So I thought let’s try these flower remedies, and within two days the dog was well.

Here the term ‘data’ may refer to a range of evidence, from digital measurement logs (such as those that self-tracking applications record) to mental records of one’s own experiences.

In this way, the participants acted as active producers and collectors of knowledge and assembled bits and pieces of information in an attempt to create a personalised or situated system of meaning (for similar results, see Broom, 2009; Pantzar and Ruckenstein, 2017). They compiled knowledge from a range of sources: medical and psychological research, but also from their personal experiences and those of friends, relatives and acquaintances, Facebook groups, blogs, websites, and books and training sessions on alternative medicine, popular psychology and new spirituality. Thus, while drawing on scientific knowledge to formulate their perceptions of health and their relationship to evidence-based recommendations, EFM users also assembled ‘social ideas, religious beliefs, situated experiences and specific worldviews’ (Wilcox, 2010: 55). Through this kind of assemblage, the participants critically assessed, redefined and diversified the knowledge base of medical recommendations and guidelines.

While many acknowledged that ‘anecdotal evidence’ from personal experience did not necessarily prove anything in a scientific sense, they sometimes tried to connect their experiential or data-based evidence with scientific evidence, as a mode of personal science and practical knowledge production (see also Heyen, 2020). Some self-trackers brought up the idea that in their interactions with medical experts, they could ‘prove’ something through data, or that in encounters with medical personnel, the data could act as an intermediary that also backs the expertise of the doctor, as doctors would not have to rely merely on what the patient says. Simultaneously, EFM practitioners may end up working on their own interpretation of experts’ evidence-based terms and sometimes also seek to transform the meaning of ‘evidence’. What is central in these instances of experimentation is the participants’ perceived adherence to the principles of scientific scepticism. Medical knowledge was not necessarily prioritised over other forms of knowledge, as long as there was some kind of perceived systematic scepticism involved, whether this scepticism manifested in digital data-based experimentation or in other forms of rationalisation through trial and error.

As with the other two forms of critique, the critique of recommendations and guidelines may also be seen as inclined towards collaboration with experts. Erika, a mother of five partly vaccinated and non-vaccinated children, was actively engaging with representatives of the National Institute for Health and Welfare, trying to work with them to find answers to questions that had not been sufficiently answered by existing scientific evidence:

I’ll ask questions if I have them, and I’ll see if they have another answer [than the one I’ve found]. I like to see what the experts think. [. . .] They do know a lot, it’s just that sometimes they leave relevant information out [when communicating about health].

However, she lamented that the representatives of health authorities would often end the discussion ‘when the questions get too tough’. In some ways, the participants’ views complied with what Giddens (1991: 3) has called the ‘institutionalisation of the principle of radical doubt’ in late modernity, which insists that ‘all knowledge takes the form of hypotheses: claims which may very well be true, but which are in principle always open to revision and may have at some point to be abandoned’. It is important to underline that this form of critique may also be interpreted as an adoption of the ideal of the neoliberal self-monitoring and self-governing health citizen (Wyatt et al., 2010), even though it sometimes manifests in forms that are deemed ‘unorthodox’ or even dangerous by official medicine.

This critique voices a willingness to participate in both macro-level and micro-level knowledge and ‘evidence’ production. Here we see a strong drive towards collaborative action, as users of EFM wish to bring their own evidence to the table. Based on the ethnographies, we do not know for sure to what extent the participants collaborate with the medical establishment, and their notions could be interpreted as attempts to demonstrate their rationality in an ethnographic interview situation. However, it seems that the participants are willing to produce material from their own experiences and data collection activities to feed into and reform recommendations and guidelines (cf. Jauho, 2016: 338). This may highlight that the issue has not aroused enough attention in the medical establishment. Also, they are eager to ask ‘wicked questions’ about evidence, and they expect to get credible answers to their concerns, suggesting that among many EFM users’ perhaps irrationalised concerns, there might be some unrecognised reasonable questions (see also Wynne, 2006: 219).

## 7. Conclusion

In this article, we have suggested the concept of EFM to bring together various critical health and well-being practices and to unravel the complex modes of contestation and appreciation of the medical establishment articulated within them. While we do not suggest that the concept of EFM should (or even could) entirely substitute for the concept of CAM, we argue that EFM allows us to address and rectify some of CAM’s problematic aspects. The first is that the concept of EFM can better capture the multiplicity of self-care practices and their varying relationships with the medical establishment than the dichotomous approach suggested by CAM. We have shown that even in instances that tend to be interpreted as ignoring science and/or spreading misinformation – accusations often levelled against vaccine-critical expressions and body–mind–spirit practices – we find active attempts to apply the scientific ideals of ethical conduct, rational scepticism and evidence-based knowledge, albeit in ways that may clash with medical knowledge. Second, EFM’s location at the boundary allows us to appreciate that the same people can be both critical and compliant simultaneously or can accept the medical establishment on some issues while opposing it on others, whereas the CAM concept – somewhat unjustly – indicates blank opposition to the medical establishment. Third, we wish to highlight that while CAM tends to foreground exceptionality, the concept of EFM captures practical and (at least from the point of view of users themselves) normal daily routines that belong to the continuum of practices that users might perform for their health. Fourth, as a much-used concept, CAM has attained some stability as a set of practices defined by the medical establishment, for example, as a ‘Medical Subject Headings’ entry term (National Library of Medicine, 2020) or a medical association’s definition of therapies that automatically fall outside the medical realm (Finnish Medical Association, 2017), whereas the concept of EFM allows us to include emerging practices and new conglomerations of activities that mix and match issues that CAM treats as incommensurate.

Thus, the concept of EFM has enabled us to shed light on the broader phenomenon of critique and contestation of the medical establishment. All three forms of critique show that people do not simply reject biomedicine but seek to appropriate its ways or ideals as part of their everyday modes of self-care. We wish to conclude by drawing broader critical conclusions on the basis of our analysis. First, we have highlighted that all the EFM domains addressed in this article afford both a critique of medical knowledge production, professional practices and medical experts’ knowledge base and a will to contribute to and improve them. Rather than waging the straightforward crusade against science which ‘non-medical’ health practices are often accused of in public debates, EFM users express a willingness to collaborate and engage with the medical establishment. This points to the need for the medical establishment to develop public engagement with its critical or ‘othered’ groups. This may be achieved by sharing the activities of medical science with the public, encouraging and listening to critical groups’ feedback regarding medical professionals and engaging with the public when formulating recommendations and guidelines based on evidence.

The latter point has consequences that need to be thought through in the context of medical knowledge production, in medical professional work and in the formulation of population-level evidence-based guidelines and recommendations. It might be beneficial to approach patients as knowledgeable subjects who are able to engage with medical knowledge. Also, it is important to acknowledge that there is knowledge production among the public of which scientists and medical practitioners may not be aware and that people are using and referring to this knowledge in their health practices. It may be important to assume that time needs to be spent on encounters with patients, and that the work of professionals needs to focus increasingly on explaining the bases of biomedical knowledge production, the professional practices themselves and the bases for scientific evidence. Medical encounters thus require dialogue, negotiation and the translation of different epistemes and systems of knowledge.

In relation to the simultaneous embrace and critique of biomedicine and its ideals, it is noteworthy that within all the EFM practices in this study, the critique also frequently turned inwards. This meant that the EFM practitioners drew moral boundaries between ‘rational critics’ and ‘irrational’ fellow practitioners. In this way, they were often involved in identity work, delineating the contours of what counts as ‘legitimate critique’. They sought to disidentify themselves from those they perceived as ‘extremists’, ‘conspiracy theorists’ or ‘amateurs’, or more generally from those whom they saw as not taking the principles of scientific scepticism and evidence-based thinking seriously, or as uncritically adopting everything they read online rather than ‘using their own brains’ and demonstrating critical thinking and an appreciation of professional knowledge. One reason for this presentation of the self as a critical subject might be the desire to demonstrate one’s potential ability to collaborate with medical researchers, professionals and experts, and thereby to gain legitimacy for EFM as a form of care. In part, this inward critique can also be seen as an attempt to further popularise, elevate and even professionalise the status of critical practices (cf. Givati and Hatton, 2015) that are often stigmatised in society.

Finally, as we have argued, rather than being ignorant about scientific principles, inattentive to societally, economically and culturally bounded mechanisms of knowledge production, or compliant with any kind of mistreatment in communications between professionals and patients, EFM users’ accounts could be interpreted as strategic action geared towards drawing the lines between good and bad practices of knowledge production and between ideal and unsuccessful patient–professional communication and towards creating a division between evidence-based and experiential knowledge. In other words, they could be interpreted as creative boundary work (Gieryn, 1999) that aims to create a space to define the medical establishment and simultaneously legitimise various EFM practices and actions. EFM users seem to be aiming to raise the status of EFM and/or gain symbolic recognition by employing the language and logic of scientific knowledge production and expert actors such that they themselves are also players in the expertise game. This boundary work and the power game related to it remain to be explored further in future research.
